# Can Tick Microbiome Explain Nonlinear Relationship between Tick Abundance and Lyme Disease Incidence?

**DOI:** 10.3390/pathogens12101229

**Published:** 2023-10-10

**Authors:** Miray Tonk-Rügen, Zbigniew Zając, Alejandro Cabezas-Cruz

**Affiliations:** 1 Institute for Insect Biotechnology, Justus Liebig University, Heinrich-Buff-Ring 26-32, 35392 Giessen, Germany; 2 Department of Biology and Parasitology, Faculty of Health Sciences, Medical University of Lublin, Radziwiłłowska 11 St., 20-080 Lublin, Poland; 3 ANSES, INRAE, Ecole Nationale Vétérinaire d’Alfort, UMR BIPAR, Laboratoire de Santé Animale, F-94700 Maisons-Alfort, France

Ticks (Acari: Ixodida) are hematophagous ectoparasitic arachnids that feed on the blood of vertebrate hosts, posing significant concern due to their unrivaled capacity to transmit various pathogens, which surpasses those of all other known arthropod vectors [1]. These pathogens include protozoa (e.g., *Babesia* spp. and *Theileria* spp.), bacteria (e.g., *Rickettsia* spp. and *Borrelia* spp.), viruses (e.g., Crimean–Congo hemorrhagic fever and tick-borne encephalitis), and helminths (e.g., *Cercopithifilaria*) [2,3,4]. 

Recent research published by Ostfeld and Keesing [5] investigates the effectiveness of various methods for reducing tick populations, specifically host-seeking nymphal blacklegged ticks. Additionally, it seeks to understand the potential impact of these tick reduction methods on human exposure to tick-borne diseases (TBDs), particularly Lyme disease. The research investigates whether reducing tick populations through acaricidal treatments and other means actually leads to a reduction in human encounters with ticks and a decrease in the incidence of TBDs in affected populations. The main conclusion of the research is that while reducing tick populations holds promise for tick control, its direct impact on reducing human encounters with ticks and TBDs is not well-established and may be influenced by various factors that require further investigation.

Environmental factors play significant roles in tick populations and pathogen prevalence [6]. Variables like temperature, humidity, and vegetation affect tick abundance and distribution [6]. Moreover, the presence of suitable hosts influences tick populations and their ability to acquire and transmit pathogens [7,8]. Changes in land use and habitat can disrupt ecological dynamics, potentially affecting TBDs prevalence in a region [9,10]. Thus, environmental factors create a complex interplay that shapes the relationship between ticks and the pathogens they carry, impacting tick-borne pathogen (TBP) transmission. The study by Ostfeld and Keesing [5] suggests that these factors result in the emergence of nonlinear dynamics between tick abundance and human exposure to TBDs. Reducing tick populations does not proportionally decrease TBDs incidence, and the mechanisms behind these nonlinearities remain poorly understood.

Modern sequencing techniques have transformed our ability to study bacterial pathogens and symbionts, opening new avenues for exploring the complex tick microbiome [11,12,13]. The tick microbiome plays a crucial role in tick physiology, host–pathogen interactions, and potentially influences pathogen transmission and disease ecology. The significance of tick microbiome for pathogen transmission cannot be overstated [12]. Could the tick microbiome and factors influencing it contribute to the nonlinear dynamics between tick abundance and human exposure to TBDs?

The tick microbiome comprises various microorganisms that can either compete with or facilitate pathogen growth and colonization [14,15,16]. For example, one study found that an increase in the proportion and quantity of the maternally inherited symbiont *Rickettsia belli* in the microbiota negatively correlates with the infection levels of the TBP *Anaplasma marginale* in *Dermacentor andersoni* ticks [17]. Regarding *Borrelia*, tick microbiome composition and diversity influence *Borrelia* acquisition in ticks [18,19]. For instance, the abundance of *Pseudomonas*, *Bacillus*, or Enterobacteriacea negatively correlates with *Borrelia burgdorferi* abundance in nymphal *Ixodes scapularis* ticks [20]. However, this relationship is not always hostile. An earlier study showed that a reduction in *Francisella*-like endosymbionts was linked to lower infection levels of *Francisella novicida* in *Dermacentor andersoni* ticks, indicating a positive pathogen–endosymbiont relationship [17]. Therefore, various factors affecting the tick microbiome can indirectly shape the disease ecology and dynamics.

Biotic factors significantly impact tick microbial communities (Figure 1). Nymph and adult ticks exhibit distinct bacterial profiles influenced by factors like tick species and geographic location [12]. Tick species are a primary influencer, with different species having unique adaptations that affect gut microbial communities [21,22,23,24]. Life stage also plays a role, with larvae, nymphs, and adults displaying different feeding behaviors and microbiome compositions [22,25,26]. Development and molting introduce changes to gut microbiome composition and diversity. Geographical locations strongly influence tick microbiome due to diverse wildlife hosts, carrying distinct microorganisms in their blood and/or skin [27,28,29,30]. Regional variations in pathogen prevalence indirectly affect tick microbiome dynamics [31,32].

Notably, ticks spend a substantial portion of their life cycle off-host in the environment [33], where they can acquire bacteria from the surrounding soil and plants [34]. Interestingly, changes in *B. burgdorferi* prevalence in ticks have been associated with factors shaping soil microbial diversity. Forest fragmentation, impacting both host and soil microbial diversity by creating new ecotone habitats [35], has been linked to higher *B. burgdorferi* prevalence in ticks [36,37]. However, the influence of land use and soil properties on the tick microbiome is an area that requires further investigation. During their off-host stages, ticks acquire bacteria, primarily from the surrounding soil and plants, through direct contact. This acquisition process involves the proteolytic activity of surrounding bacteria and fungi, which can penetrate the chitin shell of ticks via enzymatic processes [38]. Soil properties, including texture and microbial communities, likely shape the tick microbiome, impacting TBPs in ticks.

The tick microbiome has been shown to contain soil-associated bacteria. Microbiome diversity has been studied in various hard tick species, such as *Ixodes scapularis* and *Dermacentor variabilis*, using next-generation sequencing techniques [34]. Soil-associated bacteria often comprise the most diverse component of the tick microbiome [39], primarily influenced by the tick’s geographic location [40]. However, the specific impact of soil bacteria composition on the tick microbiome community, including TBPs, remains unknown.

In conclusion, the intricate world of the tick microbiome emerges as a pivotal player in the complex and nonlinear dynamics between tick abundance and human exposure to TBDs delineated by Ostfeld and Keesing [5]. As highlighted by recent research, tick microbiome wields significant influence over the physiology, behavior, and ecology of ticks, acting as key mediators in the transmission of various TBPs. The interplay between the diverse microorganisms within ticks, acquired from the environment and influenced by factors ranging from tick species to geographical locations, presents a dynamic landscape. These microorganisms can either inhibit or facilitate the transmission of TBDs, introducing a layer of complexity to the straightforward relationship between reducing tick populations and decreasing the incidence of TBDs. Understanding the nuanced role of the tick microbiome in shaping disease ecology is thus paramount, not only in comprehending the nonlinearities observed, but also for developing more targeted and effective strategies in the realm of tick control and the prevention of TBDs. Future research endeavors aiming to unravel the intricate relationships within the tick microbiome hold the promise of providing valuable insights into the broader dynamics of TBDs and refining our strategies to mitigate their impact on public health.

## Figures and Tables

**Figure 1:**
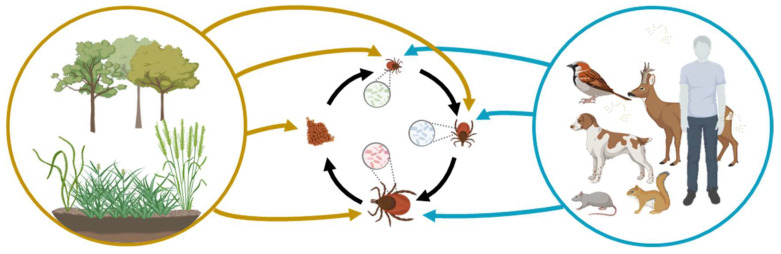
Interplay between abiotic and biotic factors in shaping the tick microbiome and disease ecology. Blue arrows indicate microorganisms obtained through tick bites. Black arrows represent maternally inherited tick symbionts acquired through transovarial and transstadial transmission. Yellow arrows signify microorganisms acquired from the environment including soil and plants (amended from a previous publication [12]). The figure was generated using BioRender.com.
